# Assessing the Risk of Contrast-Induced Nephropathy Using a Finger Stick Analysis in Recalls from Breast Screening: The CINFIBS Explorative Study

**DOI:** 10.1155/2017/5670384

**Published:** 2017-09-11

**Authors:** I. P. L. Houben, C. J. L. Y. van Berlo, O. Bekers, E. C. Nijssen, M. B. I. Lobbes, J. E. Wildberger

**Affiliations:** ^1^Department of Radiology and Nuclear Medicine, Maastricht University Medical Center, Maastricht, Netherlands; ^2^Department of Clinical Chemistry, Maastricht University Medical Center, Maastricht, Netherlands

## Abstract

**Purpose:**

To evaluate whether a handheld point-of-care (POC) device is able to predict and discriminate patients at potential risk of contrast-induced nephropathy (CIN) prior to iodine-based contrast media delivery.

**Methods and Materials:**

Between December 2014 and June 2016, women undergoing contrast-enhanced spectral mammography (CESM) with an iodine-based contrast agent were asked to have their risk of CIN assessed by a dedicated POC device (StatSensor CREAT) and a risk factor questionnaire based on national guidelines. Prior to contrast injection, a venous blood sample was drawn to compare the results of POC with regular laboratory testing.

**Results:**

A total of 351 patients were included; 344 were finally categorized as low risk patients by blood creatinine evaluation. Seven patients had a eGFR below 60 ml/min/1.73 m^2^, necessitating additional preparation prior to contrast delivery. The POC device failed to categorize six out of seven patients (86%), leading to (at that stage) unwanted contrast administration. Two patients subsequently developed CIN after 2–5 days, which was self-limiting after 30 days.

**Conclusion:**

The POC device tested was not able to reliably assess impairment of renal function in our patient cohort undergoing CESM. Consequently, we still consider classic clinical laboratory testing preferable in patients at potential risk for developing CIN.

## 1. Introduction

In the Netherlands, all women aged between 50 and 75 years are invited biennially for screening mammography. When suspect lesions are found, women are recalled to a hospital of their choice for further diagnostic testing. Contrast-enhanced spectral mammography (CESM) has been shown to be a reliable problem-solving tool in these recalled women, as it diagnoses breast cancer accurately, while ruling out breast cancer confidently [1, 2]. In addition, studies have shown that the quality of a (low-energy) CESM image is like a full-field digital mammogram (FFDM), thus omitting the necessity of performing a FFDM when a direction indication for CESM exists [3, 4]. Since CESM uses an intravenous administration of an iodine-based contrast agent, new logistical challenges must be solved [5].

Hence, patients with risk factors such as advanced age, diabetes, or heart failure, are screened by questionnaires beforehand to discriminate whether prophylactic measures should be considered prior to the exam itself.

In general, it takes one to several hours for clinical laboratory tests of renal function to become available, which will delay diagnostic testing by several hours or even by a working day. This makes the use of POC systems attractive from a workflow perspective.

Therefore, the aim of this study is to test the clinical applicability of a rapid finger stick analysis to determine renal function prior to iodinated contrast agent administration within several seconds, as opposed to hours required for a classic laboratory determination.

## 2. Materials and Methods

Women eligible for CESM in the period December 2014 to June 2016 were asked to voluntarily participate in this observational study. Exclusion criteria were known allergies for iodine-based contrast agents or contraindications to undergo CESM (such as breast implants). The local ethical committee waived the requirement for formal written informed consent (decision number METC 14-4-168).

Based on the guidelines provided by our national safety program (“VMS Veiligheidsprogramma”) the following data regarding risk factors for CIN were collected via a questionnaire [6]: type 2-diabetes, Kahler's disease, Waldenström's disease, peripheral artery disease, heart failure, anemia, hypotension, dehydration, and nephrotoxic medication. To assess serum creatinine levels and estimated glomerular filtration rate (eGFR), a point-of-care (POC) finger stick measurement (StatSensor CREAT, Nova Biomedical Corporation, Waltham, MA, USA) was performed according to the instruction manual. In short, the POC device was prepared by inserting age, race, and sex into the device, followed by the insertion of a blood sampling strip (StatStick, Nova Biomedical). Using a small finger prick, a capillary blood sample was applied onto the strip, which triggered both the automated analysis of the serum creatinine level and the calculation of the eGFR. The time needed for the analysis of eGFR and creatinine level by the device was measured.

Next, an intravenous 22 G catheter was placed in the left/right antecubital vein and venous blood samples were drawn within 15 minutes of the POC measurement using a vacuum system (Vacutainer, Becton, Dickinson and Company Europe, Eysins, Switzerland) and used for the clinical laboratory testing and were collected in a tube (with clot activator and gel for serum separation as additive) (Vacutainer with Hemoguard Gold, Becton). After venous blood sampling, automated contrast injection (Ultravist 300, Bayer Healthcare, Berlin, Germany) was performed as part of the CESM exam (dose 1.5 mL/kg body weight, flow rate 3 mL/s).

Serum creatinine was assessed using the enzymatic method (Cobas 8000; Roche Diagnostics, Rotkreuz, Switzerland). The eGFR has been calculated following the IDMS- (Isotope Dilution Mass Spectrometry-) traceable MDRD (Modification of Diet in Renal Disease) study [7]. We were not able to monitor the exact analytical time for the laboratory measurements, since these are collected in the central laboratory department and tested in batches. Also, taking blood samples to a central laboratory facility results in transportation time, which is undoubtedly much slower than a rapid POC-analysis.

Based on the different test results, patients were categorized as low or potential risk for developing CIN. The results of the POC measurements were used to determine if contrast administration was regarded as safe at this moment. The results of the laboratory served as the reference standard in our evaluation of the POC measurement. When the laboratory results categorized a patient as potential risk afterwards, additional blood analyses were performed after 2–5 days and after 30 days to check for clinical signs of CIN.

### 2.1. Statistical Analysis

For this study, descriptive statistics were used. The mean analytical time of the POC, including its standard deviation, was calculated. All analyses were performed using SPSS (IBM SPSS Statistics; version 23. IBM Corporation, Armonk, NY, USA).

## 3. Results

In the study period, 365 patients were recalled from the breast cancer screening program and volunteered for study participation. All 365 patients gave informed consent and participated in this study.

14 patients had to be excluded due to the inability to withdraw venous blood through the vacuum system used.

**Figure 1:**
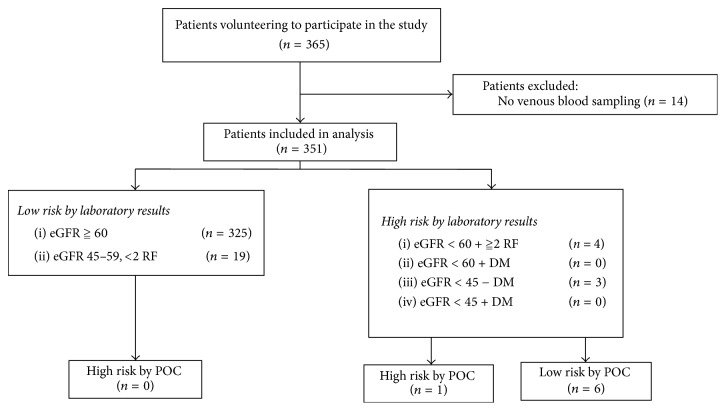
Flowchart of patient inclusion and final risk assessment categories for both of the laboratories as the point-of-care (POC) measurements. eGFR: estimated glomerular filtrations rate, RF: risk factor, and DM: diabetes.

Of the 351 included patients, 350 patients (99.7%) were categorized as low risk based on the questionnaire and POC measurement. In contrast, 344 patients (98.0%) were determined as low risk by the laboratory results. In this latter group, all patients were correctly indicated as being low risk by the POC measurement. Seven patients were determined as potential risk by the laboratory results. Of these, three patients had an eGFR < 45 ml/min/1.73 m^2^, whereas four patients had a eGFR < 60 ml/min/1.73 m^2^ with more than two risk factors. The POC device correctly identified one patient as potential risk for CIN, only (Figure 1). The POC device determined a creatinine level of 55 ml/min/1.73 m^2^; the patient had five additional clinical risk factors (age, heart failure, anemia, hypotension, and nephrotoxic medication). Despite this information, the radiologist on call decided (after consulting the referring physician) to continue with CESM nonetheless, since prehydration in this patient suffering from heart failure was expected to cause even more harm. In addition, she underwent contrast-enhanced imaging exams before using iodine-based contrast agents with no adverse effects on her renal function. Her eGFR prior to CESM was 46 ml/min/1.73 m^2^ (laboratory results) and 49 ml/min/1.73 m^2^ after 5 days.  Table 1 presents a case-by-case description of the patients that were at potential risk of developing CIN according to the laboratory results.

In contrast, six patients were indicated as low risk by the POC measurement, although the laboratory measurements in combination with various risk factors classified these patients as potential risk. Hence, these patients incorrectly received contrast agent based to the POC measurements and were subsequently recalled for an additional blood sampling. CIN was diagnosed in two patients, with renal function normalization after 30 days.

The mean analytical time for a POC measurement was 47.8 seconds (SD 5.1 seconds), which is without doubt much faster than any clinical laboratory testing.

## 4. Discussion

CIN is an important side-effect of the administration of iodine-based contrast agents, with a reported incidence from 1 to 30%, depending on the population studied [7]. In the assessment of risk of developing CIN, measurement of renal function (i.e., serum creatinine levels) plays a pivotal role. However, measurement of serum creatinine levels in clinical laboratories takes often a minimum of one hour to perform, which is unwanted in scenarios where speedy diagnostics are preferred.

In this study, we aimed to test the clinical applicability of a rapid finger stick analysis to determine renal function prior to iodinated contrast agent administration in exams which are logistically challenging, such as CESM on an outpatient basis.

For this purpose, rapid POC measurements are available that can assess renal function within a much shorter time frame (mean time in our study is 48 seconds). These usually consist of handheld devices in which applicator strip is inserted which can analyze a small drop of blood acquired through a small finger prick. Martínez Lomakin and Tobar recently reviewed a larger number of currently available POC devices and concluded that these in general suffered from a moderate concordance when compared to standardized renal function measurements [8]. To be more specific: these devices have a small mean difference in measurements when compared to standard methods, but their 95% limits of agreement often lay between −35.4 and 35.4 *µ*mol/L, sometimes even exceeding 88.4 *µ*mol/L. This could lead to an important number of false-negative results by POC measurements, exposing patients to iodine-based contrast agents when they are at potential risk of developing CIN. However, the findings of Martínez Lomakin and Tobar are difficult to translate to the clinical setting, since multiple devices were used in the different studies, which also differed in populations studied and reference standards used [8].

In our study, most patients were at low risk for developing CIN and correctly identified by POC measurements. However, the smaller number of patients who were at potential risk for developing CIN could not be identified by using the POC measurement: only one out of seven potential risk patients was correctly identified. One patient might be regarded as borderline normal with an eGFR of 59 ml/min/1.73 m^2^ and a POC value of 63 ml/min/1.73 m^2^, which is within the error limit of any diagnostic test. However, the other five patients must be regarded as a knock-out for the clinical applicability of the device tested. These patients incorrectly received contrast administration, of which two developed CIN after several days. CIN was self-limiting in all patients within 30 days. Thus, when performing CESM in daily practice, the clinical pathway will have to follow the current questionnaire assessment. If triggers for CIN are found, an intravenous blood sample will have to be drawn, regardless of the time needed for analysis, to allow for adequate risk assessment for CIN prior to contrast material delivery.

Of note, the clinical impact of CIN is still under debate. A recent retrospective study of postcontrast acute kidney injury after CT exams showed that the odds ratio for developing acute kidney injury starts to increase from eGFR levels of 30–44 ml/min/1.73 m^2^, with the highest odds ratio (OR 2.96) in patients with eGFR < 30 ml/min/1.73 m^2^. In contrast, a similar study by McDonald et al. found no such increased risk in this latter patient category [9–11]. Since CIN cannot be treated, many studies and national guidelines have focused on its prevention, most commonly by using prehydration protocols for patients at risk for CIN. In this regard, it was recently shown that refraining from prehydration is noninferior and cost-saving in preventing CIN compared with preventive prehydration [12]. The current study, however, was based on current national guidelines and focused on finding a new (POC-based) strategy to deal with logistical challenges caused by these guidelines. Whether the most recent insights are a cause to reevaluate the currently existing national guidelines regarding the prevention of CIN is beyond the scope of this paper.

Our study has some limitations. First, we only included patients scheduled to undergo CESM for a screen-recalled breast lesion. In our country, these are women between 50 and 75 years with a low prevalence of risk factors for developing CIN. In previous studies, the concordances between the POC measurements and the reference standard decreased with increased serum creatinine levels [8]. This might further limit the utility of these devices in patients with a higher chance of developing CIN, that is, patients with more risk factors, such as cardiac patients or patients from an intensive care unit. Hence, even though the population studied was a specific one, it contained patients with the most favorable profiles for the best performance of the POC device. Second, only women recalled from screening that underwent CESM were asked to participate in this observational study. We think they are quite representative for the general population of a breast imaging department, that is, women over 50 years of age, the majority being postmenopausal. Also, we tested only a single POC device. Thus, one should interpret our observations with these limitations in mind.

In summary, the clinical use of POC systems is attractive, especially considering workflow logistics. From a patient safety perspective, however, the handheld POC device tested (StatSensor CREAT) was not able to reliably assess impairment of renal function in our patient cohort undergoing CESM. Consequently, we consider classic clinical laboratory testing preferable in patients at potential risk for developing CIN.

## Figures and Tables

**Table 1:** Detailed characteristics of the patients at high risk for developing contrast-induced nephropathy (CIN). eGFR: estimated glomerular filtration rate, POC: point-of-care measurement, LAB: clinical laboratory measurement, DM: diabetes mellitus type II, PAD: peripheral artery disease, HF: heart failure, HT: hypotension, DH: dehydration, Med: nephrotoxic medication, IDC: invasive ductal carcinoma, and nos: not otherwise specified. Unit of eGFR measurement: ml/min/1.73 m^2^. Patient #4 was the only correctly identified high risk patient as determined by the POC handheld device.

Patient	Age (yr)	eGFR POC	eGFR LAB	DM	Kahler	Waldenström	PAD	HF	Anemia	HT	DH	Med	CIN	Pathology
1	57	47	43	No	No	No	No	No	No	No	No	No	No	Duct ectasia
2	57	60	40	No	No	No	Yes	Yes	No	No	No	No	Yes	Apocrine changes
3	69	63	59	No	No	No	No	No	No	Yes	No	Yes	No	IDC
4	74	55	46	No	No	No	No	Yes	Yes	Yes	No	Yes	No	Benign nos
5	65	78	52	No	No	No	No	No	No	Yes	No	Yes	No	Cyst
6	73	90	58	No	No	No	No	No	No	Yes	No	Yes	Yes	Duct ectasia
7	71	60	37	No	No	No	No	No	No	No	No	No	No	Cyst
